# Supplementary material for: A Likelihood Approach for Real-Time Calibration of Stochastic Compartmental Epidemic Models
Source: PLoS Comput Biol. 2017 Jan 17;13(1):e1005257. doi: 10.1371/journal.pcbi.1005257 (PMC5240920; doi:10.1371/journal.pcbi.1005257)
Supplement: S1 File — (TAR.GZ) [file pcbi.1005257.s014.tar.gz › HSPH_Online-SI-Revision/MSS10/n1-mild/n1-mild_NEW_table4.pdf]

1	R0	{1.27677, 7.89375, 1.}	{1.27085, 5.95308, 2.03}	{{1.24271, 5.40945, 2.}, {1.34668, 8.42466, 3.}}	{2.50129, 26.7861, 113.}	inf	18.	17.9039	{6.92012, 30.9046}	42.0259	0.206946	newly	14.	19.09	{7., 29.}	59.6429	0.227388	Reff	0.891185	0.886897	{0.865658, 0.917419}	2.00162	ar	400.	381.89	{305., 473.}	13.0525
2	R0	{1.33231, 10.5259, 2.}	{1.28606, 3.81199, 0.09}	{{1.25474, 3.26445, 0.}, {1.28596, 3.98513, 0.}}	{4.29147, 63.7846, 95.5}	inf	9.	4.57	{1.88717, 7.28159}	50.0587	0.354103	newly	6.	6.38	{2., 12.}	49.	0.227877	Reff	0.735435	0.715586	{0.696613, 0.719642}	3.57539	ar	467.	454.85	{442., 474.}	3.98501
3	R0	{1.2273, 6.76944, 1.}	{1.11976, 2.41628, 0.03}	{{1.06738, 2.19254, 0.}, {1.24751, 3.07157, 0.}}	{9.29189, 64.3061, 97.}	inf	10.	7.54772	{1.98994, 13.814}	46.802	0.293293	newly	13.	17.85	{4., 34.}	79.4615	0.30265	Reff	0.924157	0.845916	{0.799663, 0.937273}	9.17785	ar	314.	280.24	{241., 344.}	16.3248
4	R0	{1.41215, 17.5742, 3.}	{1.12498, 2.84045, 0.32}	{{1.06256, 2.19254, 0.}, {1.25474, 3.26445, 1.}}	{20.3359, 83.8374, 89.3333}	inf	22.	6.27574	{1.93682, 11.5042}	71.4739	0.635456	newly	12.	13.86	{3., 27.}	61.5	0.287282	Reff	1.14808	0.932244	{0.881427, 1.03247}	18.7996	ar	465.	218.37	{168., 297.}	53.0387
5	R0	{1.42222, 6.15886, 0.1}	{1.2738, 2.82677, 0.}	{{1.22544, 2.5364, 0.}, {1.39162, 3.26445, 0.}}	{11.0428, 54.1024, 100.}	inf	19.	8.04514	{2.38018, 14.4365}	57.8372	0.460483	newly	13.	14.27	{3., 25.}	55.3077	0.274191	Reff	0.797865	0.728512	{0.703473, 0.798725}	9.5771	ar	490.	445.25	{424., 468.}	9.31224
6	R0	{1.24363, 9.95596, 0.1}	{1.25167, 3.48492, 0.1}	{{1.21159, 3.07157, 0.}, {1.28596, 3.98513, 0.}}	{2.7284, 64.9966, 182.}	inf	38.	19.1513	{5.85788, 33.7709}	50.3036	0.38085	newly	28.	36.45	{11., 67.}	66.5357	0.264007	Reff	0.93521	0.964846	{0.927756, 1.00174}	4.12144	ar	411.	342.45	{226., 448.}	23.0389
7	R0	{1.36478, 5.96056, 1.}	{1.39073, 1.89344, 0.03}	{{1.39162, 1.89883, 0.}, {1.39162, 1.89883, 0.}}	{1.99311, 68.2339, 101.}	inf	20.	19.4919	{3.21092, 39.1001}	55.4417	0.304566	newly	19.	53.28	{9., 103.}	199.684	0.45427	Reff	0.848893	0.865757	{0.836499, 0.88723}	2.74574	ar	484.	446.08	{369., 532.}	12.5372
8	R0	{1.36335, 17.6429, 2.}	{1.15157, 5.69447, 0.62}	{{1.10202, 5.30671, 0.}, {1.18976, 6.0993, 2.}}	{15.5337, 67.7237, 69.}	inf	41.	13.6504	{4.46083, 21.742}	66.7062	0.540146	newly	19.	16.25	{6., 26.}	37.8421	0.218623	Reff	1.08932	0.951522	{0.915336, 0.978678}	12.6496	ar	484.	261.63	{174., 353.}	45.9442
9	R0	{1.26575, 5.28707, 0.1}	{1.38288, 3.94757, 0.07}	{{1.28596, 3.83841, 0.}, {1.47488, 4.06661, 0.}}	{9.48553, 25.3354, 162.}	inf	24.	12.6538	{3.05891, 22.5755}	50.3915	0.386541	newly	28.	18.73	{3., 33.}	44.1786	0.319496	Reff	0.731603	0.814847	{0.746842, 0.868008}	11.4241	ar	472.	449.7	{407., 497.}	8.14831
10	R0	{1.42942, 9.49905, 2.}	{1.39956, 8.26611, 0.06}	{{1.39882, 8.26611, 0.}, {1.39882, 8.26611, 0.}}	{2.08925, 12.9796, 97.}	inf	17.	24.8794	{14.141, 36.0592}	54.2135	0.186053	newly	11.	19.03	{10., 25.}	79.	0.244164	Reff	0.773316	0.746142	{0.729976, 0.756877}	3.61282	ar	468.	526.57	{486., 574.}	12.6346
11	R0	{1.46827, 15.0983, 0.1}	{1.2018, 8.33825, 1.05}	{{1.17082, 5.50828, 1.}, {1.21937, 10.1856, 2.}}	{18.1483, 44.7736, 964.}	inf	17.	25.8844	{7.96907, 41.839}	77.5012	0.273279	newly	6.	20.91	{10., 31.}	250.5	0.516596	Reff	1.17902	0.954195	{0.938252, 0.977534}	19.0689	ar	495.	326.44	{218., 433.}	34.0525
12	R0	{1.25751, 13.279, 1.}	{1.09349, 2.39821, 0.1}	{{1.06738, 2.19254, 0.}, {1.22544, 3.07157, 0.}}	{13.0883, 81.9399, 92.}	inf	33.	8.0839	{2.73648, 14.3684}	75.5033	0.702934	newly	20.	20.99	{4., 44.}	61.05	0.30789	Reff	1.01481	0.909686	{0.881259, 1.01741}	10.8314	ar	366.	222.84	{166., 317.}	39.9672
13	R0	{1.3425, 9.16361, 1.}	{1.36862, 5.71455, 1.5}	{{1.34436, 4.00571, 0.}, {1.44762, 6.25107, 2.}}	{2.07648, 37.6387, 88.}	inf	14.	7.80316	{3.09606, 12.0358}	47.6428	0.329933	newly	8.	8.77	{3., 15.}	50.875	0.244262	Reff	0.765225	0.788563	{0.76928, 0.832757}	3.15778	ar	442.	447.57	{422., 483.}	4.45928
14	R0	{1.18936, 6.20583, 0.1}	{1.31421, 5.17085, 1.54}	{{1.27231, 4.94504, 1.}, {1.3618, 5.40945, 2.}}	{10.4971, 17.0762, 1454.}	inf	14.	13.3101	{4.90585, 21.4841}	35.8476	0.1874	newly	10.	15.86	{7., 27.}	73.2	0.235932	Reff	0.752865	0.832774	{0.799111, 0.871051}	10.6141	ar	382.	412.98	{367., 472.}	9.8377
15	R0	{1.27137, 9.78868, 0.1}	{1.29063, 4.05275, 0.23}	{{1.28596, 3.98513, 0.}, {1.29927, 4.21286, 1.}}	{1.66114, 58.5976, 302.}	inf	28.	15.7436	{5.22152, 25.5579}	45.443	0.333617	newly	19.	23.59	{7., 39.}	56.6842	0.240953	Reff	0.827662	0.855993	{0.837864, 0.871142}	3.46242	ar	496.	394.45	{335., 458.}	20.9294
16	R0	{1.23102, 13.6452, 1.}	{1.35864, 13.1039, 2.87}	{{1.37104, 13.5726, 3.}, {1.37104, 13.5726, 3.}}	{11.0166, 3.96707, 189.}	inf	35.	22.2108	{16.0104, 28.0797}	36.5834	0.216119	newly	16.	11.49	{7., 16.}	30.5625	0.177432	Reff	0.923265	1.03619	{1.03632, 1.05127}	12.481	ar	445.	416.09	{286., 519.}	17.5034
17	R0	{1.27213, 13.7452, 3.}	{1.15307, 5.86305, 0.59}	{{1.10202, 5.30671, 0.}, {1.18976, 6.0993, 2.}}	{9.60366, 57.4921, 80.3333}	inf	29.	14.261	{4.79265, 24.1864}	52.6062	0.390999	newly	12.	16.35	{5., 28.}	65.25	0.248709	Reff	1.02661	0.94736	{0.911949, 0.981148}	7.89435	ar	367.	266.95	{177., 377.}	30.0572
18	R0	{1.25318, 11.2151, 0.1}	{1.23145, 6.3751, 1.86}	{{1.17082, 5.40945, 1.}, {1.27231, 8.10487, 2.}}	{2.13505, 43.1561, 1766.}	inf	28.	12.7917	{4.82048, 21.5365}	54.3152	0.395331	newly	18.	13.29	{5., 20.}	33.7222	0.214396	Reff	0.917328	0.920161	{0.878587, 0.951555}	1.76414	ar	416.	323.69	{258., 410.}	23.3486
19	R0	{1.46006, 8.11496, 0.1}	{1.28179, 3.90424, 0.08}	{{1.25474, 3.26445, 0.}, {1.28596, 3.98513, 0.}}	{12.2096, 51.8883, 168.}	inf	28.	9.86115	{3.2554, 16.9462}	64.7816	0.531209	newly	24.	14.49	{5., 24.}	47.0417	0.335095	Reff	0.861435	0.7795	{0.763128, 0.789688}	9.51147	ar	496.	424.22	{388., 476.}	14.629
20	R0	{1.14273, 6.71261, 0.1}	{1.26654, 3.71572, 0.1}	{{1.21159, 3.07157, 0.}, {1.28596, 3.98513, 0.}}	{10.8344, 44.6456, 180.}	inf	18.	11.1704	{3.71521, 18.7742}	42.0808	0.295533	newly	18.	19.03	{5., 35.}	47.2778	0.2248	Reff	0.787341	0.881253	{0.850379, 0.901309}	11.9278	ar	362.	358.76	{302., 435.}	11.9558
21	R0	{1.42432, 8.23905, 1.}	{1.24382, 2.85749, 0.}	{{1.22544, 2.5364, 0.}, {1.25474, 3.26445, 0.}}	{12.6727, 65.3178, 100.}	inf	21.	9.44134	{2.60146, 16.962}	55.5214	0.43136	newly	16.	18.95	{4., 34.}	57.4375	0.257791	Reff	0.914413	0.812913	{0.797967, 0.824619}	11.1	ar	405.	381.07	{343., 433.}	9.30617
22	R0	{1.29386, 17.863, 2.}	{1.3847, 12.1642, 1.91}	{{1.29096, 10.0371, 2.}, {1.50403, 15.3523, 2.}}	{7.50509, 32.6295, 4.5}	inf	28.	36.2856	{16.8333, 61.4299}	52.9771	0.187482	newly	12.	20.88	{13., 29.}	76.8333	0.231039	Reff	1.06614	1.12759	{1.06276, 1.20743}	6.12066	ar	488.	480.38	{310., 646.}	21.2787
23	R0	{1.39274, 13.0167, 5.}	{1.28426, 3.95472, 0.02}	{{1.28596, 3.98513, 0.}, {1.28596, 3.98513, 0.}}	{7.78916, 69.6181, 99.6}	inf	35.	11.7065	{3.43682, 19.4046}	66.5528	0.559141	newly	27.	18.2	{5., 32.}	42.4444	0.313187	Reff	0.891354	0.851835	{0.83867, 0.863016}	4.43353	ar	475.	381.92	{333., 438.}	19.8442
24	R0	{1.32178, 15.8991, 1.}	{1.2732, 6.79405, 1.51}	{{1.22544, 3.98513, 0.}, {1.29991, 8.04006, 2.}}	{4.68248, 58.3113, 75.}	inf	48.	18.5415	{6.13754, 28.8398}	61.3719	0.470929	newly	20.	18.93	{8., 31.}	35.35	0.179886	Reff	0.896167	0.900539	{0.868037, 0.927514}	2.65802	ar	471.	387.55	{312., 470.}	19.7856
25	R0	{1.37568, 17.8427, 1.}	{1.27476, 9.273, 1.82}	{{1.17082, 5.77111, 1.}, {1.29096, 10.0371, 2.}}	{7.336, 48.0292, 92.}	inf	27.	27.5698	{10.0946, 38.033}	28.4841	0.1478	newly	11.	19.81	{12., 27.}	83.7273	0.251882	Reff	1.05377	0.975459	{0.922051, 0.994761}	7.43154	ar	398.	389.42	{263., 493.}	15.9799
26	R0	{1.20993, 6.4656, 0.1}	{1.25571, 6.52798, 1.95}	{{1.24271, 5.50828, 2.}, {1.25721, 6.78013, 2.}}	{3.78385, 7.0132, 1854.}	inf	9.	14.774	{7.2996, 22.5405}	75.1375	0.243298	newly	13.	14.54	{8., 22.}	34.4615	0.153244	Reff	0.834852	0.859184	{0.848134, 0.866694}	2.9146	ar	345.	374.98	{322., 433.}	11.8957
27	R0	{1.20548, 13.8003, 0.1}	{1.21599, 4.6372, 0.8}	{{1.17082, 3.07157, 0.}, {1.28596, 6.0993, 2.}}	{3.48035, 66.3978, 784.}	inf	30.	11.018	{5.0471, 16.8609}	63.2732	0.491939	newly	10.	16.36	{6., 26.}	81.	0.243883	Reff	0.876384	0.907063	{0.867505, 0.956637}	4.39474	ar	350.	321.73	{256., 406.}	16.2543
28	R0	{1.3176, 14.6594, 1.}	{1.40886, 9.79545, 0.48}	{{1.21159, 3.07157, 0.}, {1.59546, 13.1646, 1.}}	{13.3187, 34.5521, 68.}	inf	22.	27.075	{7.57262, 40.3921}	48.0453	0.223138	newly	16.	22.03	{11., 33.}	51.0625	0.171221	Reff	1.11732	1.18627	{1.03876, 1.34452}	12.5323	ar	362.	464.89	{225., 635.}	44.5497
29	R0	{1.28202, 6.23047, 1.}	{1.26313, 3.5293, 0.06}	{{1.22544, 3.07157, 0.}, {1.28596, 3.98513, 0.}}	{1.75219, 43.3542, 100.}	inf	11.	11.0272	{3.89137, 19.9093}	45.2622	0.229781	newly	14.	19.03	{6., 33.}	68.2143	0.265067	Reff	0.844851	0.832377	{0.810237, 0.853782}	2.01784	ar	391.	380.86	{337., 434.}	8.24041
30	R0	{1.28177, 8.83252, 1.}	{1.26409, 5.29563, 1.85}	{{1.24271, 3.98513, 0.}, {1.28596, 5.50828, 2.}}	{1.71271, 40.0439, 105.}	inf	22.	15.028	{4.97511, 24.5535}	38.8796	0.256443	newly	11.	18.33	{6., 32.}	86.0909	0.268466	Reff	0.848532	0.845618	{0.826535, 0.864632}	1.49179	ar	427.	387.4	{325., 472.}	13.1569
31	R0	{1.35886, 5.97708, 0.1}	{1.42034, 5.86034, 0.38}	{{1.38815, 4.00571, 0.}, {1.47488, 6.71734, 1.}}	{4.52463, 15.1504, 440.}	inf	14.	15.0579	{7.40112, 23.5527}	33.7622	0.156406	newly	13.	15.68	{8., 24.}	44.3077	0.176428	Reff	0.712043	0.742746	{0.724446, 0.77051}	4.31203	ar	491.	513.18	{479., 548.}	5.64969
32	R0	{1.27601, 17.9804, 3.}	{1.19212, 7.88251, 0.75}	{{1.06738, 2.19254, 0.}, {1.22544, 10.1856, 1.}}	{6.6056, 56.1605, 75.}	inf	32.	12.2669	{4.76357, 18.2038}	61.666	0.474696	newly	12.	12.12	{6., 21.}	41.8333	0.186592	Reff	1.03995	0.994983	{0.896565, 1.02903}	4.59016	ar	445.	266.8	{165., 390.}	40.0539
33	R0	{1.3368, 11.3235, 0.1}	{1.38062, 8.23831, 0.11}	{{1.38427, 8.35044, 0.}, {1.38427, 8.35044, 0.}}	{3.50741, 27.2459, 200.}	inf	41.	42.1166	{32.1907, 51.9723}	17.0385	0.0782714	newly	18.	33.72	{25., 43.}	88.6667	0.267615	Reff	0.838174	0.864013	{0.851448, 0.876782}	3.12151	ar	417.	505.08	{424., 566.}	21.9904
34	R0	{1.21021, 11.3635, 2.}	{1.13865, 5.30523, 0.17}	{{1.10202, 3.07157, 0.}, {1.17082, 6.0871, 1.}}	{6.48789, 53.3134, 91.5}	inf	19.	8.00017	{2.53206, 14.5425}	57.9586	0.451968	newly	11.	10.74	{3., 20.}	55.2727	0.283247	Reff	0.958486	0.914332	{0.87741, 0.94439}	5.39613	ar	327.	244.99	{195., 316.}	26.7187
35	R0	{1.53382, 10.8866, 0.1}	{1.42916, 10.6245, 1.23}	{{1.39882, 8.26611, 0.}, {1.44709, 12.3743, 2.}}	{6.82332, 17.4401, 1194.}	inf	49.	55.5764	{37.3878, 70.693}	25.7979	0.105677	newly	34.	34.95	{26., 44.}	17.4412	0.0758116	Reff	0.963239	0.887893	{0.874657, 0.903001}	7.82213	ar	476.	536.64	{467., 606.}	14.1765
36	R0	{1.20423, 6.83844, 0.1}	{1.2696, 3.58928, 0.02}	{{1.24751, 2.5364, 0.}, {1.28596, 3.98513, 0.}}	{5.42843, 47.5132, 118.}	inf	19.	10.0997	{3.37956, 18.7447}	48.7578	0.370753	newly	18.	16.09	{4., 32.}	48.7222	0.278785	Reff	0.714108	0.764175	{0.744552, 0.781774}	7.01101	ar	416.	423.93	{394., 466.}	5.32933
37	R0	{1.43629, 10.1548, 3.}	{1.26261, 4.55425, 0.7}	{{1.22544, 3.07157, 0.}, {1.31737, 10.8592, 4.}}	{12.0925, 57.0939, 86.6667}	inf	18.	12.3631	{4.13121, 21.8893}	45.053	0.307188	newly	19.	18.04	{4., 33.}	45.4737	0.264812	Reff	1.01977	0.903442	{0.868901, 0.930631}	11.4069	ar	367.	346.59	{279., 429.}	14.891
38	R0	{1.37504, 8.93428, 1.}	{1.25225, 3.19064, 0.02}	{{1.22544, 2.5364, 0.}, {1.28596, 3.98513, 0.}}	{8.92995, 64.2876, 100.}	inf	26.	9.21917	{2.80565, 15.9065}	64.5416	0.540288	newly	22.	15.64	{4., 30.}	46.7273	0.335865	Reff	0.764522	0.717264	{0.700402, 0.733142}	6.18141	ar	483.	449.08	{423., 484.}	8.21946
39	R0	{1.51637, 9.36742, 1.}	{1.28724, 4.89376, 1.39}	{{1.27231, 3.98513, 0.}, {1.3618, 5.40945, 2.}}	{15.1105, 47.7576, 93.}	inf	18.	9.84108	{2.1726, 16.6898}	46.0446	0.353335	newly	14.	11.95	{3., 21.}	39.9286	0.261103	Reff	0.938633	0.807308	{0.787984, 0.843701}	13.9911	ar	441.	406.76	{369., 454.}	9.98186
40	R0	{1.23658, 9.82869, 1.}	{1.26555, 3.38058, 0.01}	{{1.22544, 2.5364, 0.}, {1.28596, 3.98513, 0.}}	{2.73963, 65.6049, 99.}	inf	25.	13.266	{4.78543, 21.4019}	47.9023	0.344941	newly	12.	21.77	{7., 37.}	97.25	0.288699	Reff	0.746894	0.779182	{0.755468, 0.796092}	4.366	ar	414.	418.83	{380., 463.}	6.09903
41	R0	{1.24247, 5.4242, 0.1}	{1.25077, 2.48401, 0.02}	{{1.24751, 2.5364, 0.}, {1.24751, 2.5364, 0.}}	{1.51162, 54.2051, 118.}	inf	10.	12.6874	{2.75723, 24.4664}	71.336	0.301875	newly	10.	30.33	{4., 64.}	225.5	0.473784	Reff	0.861032	0.863367	{0.84523, 0.874926}	2.00055	ar	345.	367.63	{301., 459.}	14.3855
42	R0	{1.28208, 4.40223, 0.1}	{1.24246, 2.51807, 0.}	{{1.24751, 2.5364, 0.}, {1.24751, 2.5364, 0.}}	{3.60293, 42.8002, 100.}	inf	0.1	4.06218	{1.38218, 7.25495}	3962.18	1.5435	newly	0.1	9.32	{2., 18.}	9220.	1.81922	Reff	0.847455	0.816195	{0.814604, 0.822928}	4.13963	ar	339.	356.21	{341., 381.}	5.0767
43	R0	{1.56773, 6.50474, 0.1}	{1.44922, 4.04776, 0.13}	{{1.44762, 3.83841, 0.}, {1.47608, 4.06661, 0.}}	{7.5596, 37.7721, 214.}	inf	12.	10.8436	{4.03142, 17.8498}	38.7123	0.213676	newly	11.	15.32	{4., 26.}	66.5455	0.256721	Reff	0.810516	0.750912	{0.735074, 0.770284}	7.35385	ar	498.	507.91	{479., 541.}	3.98594
44	R0	{1.10489, 6.01941, 1.}	{1.2346, 3.02623, 0.}	{{1.22544, 2.5364, 0.}, {1.25474, 3.26445, 0.}}	{11.8753, 49.7255, 100.}	inf	6.	7.45066	{1.96971, 13.7442}	62.1085	0.251188	newly	6.	14.72	{3., 30.}	164.	0.386083	Reff	0.734752	0.819227	{0.806358, 0.836051}	11.6669	ar	346.	362.41	{333., 414.}	6.96821
45	R0	{1.25888, 14.1926, 1.}	{1.34853, 4.79355, 0.9}	{{1.28596, 3.98513, 0.}, {1.3618, 4.98002, 1.}}	{7.12138, 66.225, 24.}	inf	40.	12.3388	{3.89448, 21.4698}	69.153	0.588992	newly	16.	16.27	{4., 29.}	47.9375	0.251846	Reff	0.723856	0.812704	{0.777271, 0.832621}	12.2742	ar	471.	438.26	{394., 503.}	9.77919
46	R0	{1.45292, 16.616, 2.}	{1.16819, 3.75155, 0.58}	{{1.06738, 2.19254, 0.}, {1.28596, 5.30671, 2.}}	{19.5973, 77.4221, 71.}	inf	41.	8.93583	{2.42651, 15.1907}	78.5605	0.788475	newly	19.	14.84	{4., 30.}	51.6842	0.333259	Reff	1.11003	0.929701	{0.855197, 1.02199}	16.2455	ar	494.	259.16	{200., 366.}	47.9919
47	R0	{1.22528, 16.6033, 0.1}	{1.15704, 6.47315, 0.59}	{{1.13227, 5.71232, 0.}, {1.19093, 6.83219, 1.}}	{5.70041, 61.439, 600.}	inf	24.	13.1206	{5.16115, 22.5431}	48.8189	0.330678	newly	5.	14.21	{6., 23.}	189.8	0.428761	Reff	1.06599	1.01911	{0.990623, 1.05149}	4.45216	ar	368.	244.28	{137., 385.}	36.9837
48	R0	{1.1669, 6.92715, 0.1}	{1.52742, 17.254, 4.25}	{{1.39162, 2.5364, 0.}, {1.56495, 19.9037, 5.}}	{30.8959, 169.381, 4180.}	inf	8.	92.1664	{10.6611, 113.532}	1060.49	1.00489	newly	4.	34.21	{24., 43.}	756.25	0.899957	Reff	0.768987	0.874849	{0.84789, 0.892751}	13.7664	ar	416.	625.39	{403., 703.}	53.2236
49	R0	{1.32749, 16.0887, 3.}	{1.3329, 10.5763, 0.97}	{{1.33937, 10.7271, 1.}, {1.33937, 10.7271, 1.}}	{1.32817, 34.2623, 67.6667}	inf	24.	30.0916	{21.6032, 39.8013}	31.7596	0.120172	newly	12.	20.28	{14., 27.}	71.	0.222376	Reff	1.11376	1.11008	{1.10166, 1.12481}	1.00855	ar	353.	437.26	{343., 536.}	29.7677
50	R0	{1.29636, 15.2609, 0.1}	{1.25683, 6.29819, 1.95}	{{1.24271, 5.40945, 2.}, {1.27231, 6.78013, 2.}}	{3.04896, 58.7299, 1856.}	inf	49.	12.5254	{5.3211, 19.3945}	74.4379	0.638013	newly	23.	12.87	{5., 20.}	46.1304	0.319092	Reff	0.81541	0.836386	{0.825051, 0.847312}	2.58283	ar	470.	378.76	{336., 423.}	19.8383
